# Diagnostic antigens for visceral leishmaniasis: clarification of nomenclatures

**DOI:** 10.1186/s13071-017-2120-x

**Published:** 2017-04-13

**Authors:** Tapan Bhattacharyya, Tegwen Marlais, Michael A. Miles

**Affiliations:** Department of Pathogen Molecular Biology, Faculty of Infectious and Tropical Diseases, London School of Hygiene and Tropical Medicine, London, United Kingdom

**Keywords:** *Leishmania*, Visceral leishmaniasis, Serology, Antigens, Kinesin, Nomenclature, HASPB, Diagnostics, rK39, rK28

## Abstract

**Background:**

Stimulated by the increasing recent use of ‘K’ or ‘rK’ nomenclature for antigens reported for visceral leishmaniasis (VL) diagnostic serology, we wished to give a chronological synopsis of their reporting and the potentially confusing terminology.

**Methods:**

The literature was examined for ‘K' or ‘rK’ terminology for VL diagnostic antigens, with emphasis on the original publications in which terms were first used.

**Results:**

A chronological account of the first use of these 'K' and 'rK' nomenclatures was compiled. Since the original use of this terminology in 1993 in the name rK39 for a *Leishmania* antigen fragment, we found nine subsequent instances where ‘K' or ‘rK’ have been used to maintain consistency with this nomenclature. We also found instances where there were ambiguities regarding reported strain name, origin and GenBank accession numbers.

**Conclusions:**

We have documented here the uses in the literature of the ‘K’ or ‘rK’ prefix for VL diagnostic antigen nomenclature. We suggest that, to avoid confusion, the use of such nomenclature for future antigens should either provide the logical derivation of the term or indicate that the designation is entirely empirical.

## Background

We wish to clarify the potentially confusing ‘K’ or ‘rK’ nomenclature of the antigens used for visceral leishmaniasis (VL) diagnostic serology, by giving a synopsis of their discovery and naming. This has been stimulated by the increasing recent use of this terminology, as listed in Table 1. We therefore focus here on a chronological account of the first use of these nomenclatures rather than an assessment of the use of the antigens in serology or their native function, which are beyond the scope of the current article. By way of introduction it is pertinent to note that Kuhls et al. [1] demonstrated that *Leishmania chagasi*, the name that had been used for the agent of VL in South America, is synonymous with *L. infantum* deriving from Europe. Thus, the term *L. infantum* (syn. *chagasi*) will be used where appropriate.

**Table 1:** Chronology of the naming of ‘K’ or ‘rK’ antigens used for serological diagnosis of visceral leishmaniasis

Year	Reference	Antigen	Species	Parent protein	Reported origin	Strain details	GenBank accession number
1993	Burns et al. [2]	rK39	*L. infantum* (syn*. L. chagasi*)	LcKin (kinesin-related)	Brazil	MHOM/BR/82/BA-2,C1	L07879
1994	Tolson et al. [3]	KMP-11	*L. donovani*	Kinetoplastid membrane protein	Not given	LD3, derivative of 1S2D clone^a^	S77039^a^
1999	Alce et al. [4]	HASPB1/HASPB2	*L. donovani*	HASP	Ethiopia	MHOM/ET/67/L28 isolate LV9	AJ011810/ AJ011809
1999	Bhatia et al. [5]	K9/K26	*L. infantum* (syn*. L. chagasi*)	Hydrophilic protein (see text)	Brazil	MHOM/BR/74/PP75	AF131227/ AF131228
2006	Sivakumar et al. [6]	Ld-rKE16	*L. donovani*	Kinesin	India	MHOM/IN/98/KE16	AY615886
2007	Gerald et al. [7]	LdK39	*L. donovani*	Kinesin LdK39	Sudan	MHOM/SD/62/1S-CL2D	DQ831678
2007	Takagi et al. [9]	rKRP42	*L. donovani*	Kinesin	India/Bangladesh	MHOM/IN/80/DD8	AB256033
2010	Pattabhi et al. [8]	rK28	*L. donovani*	HASPB1/LdK39/ HASPB2	Sudan/Ethiopia	Synthetic gene	HM594686
2013	Abass et al. [10]	rKLO8	*L. donovani*	Kinesin	Sudan	Lo8	KC788285
2015	Vallur et al. [11]	rK18	Not given	Not given	Not given	Not given	Not given
2016	Vallur et al. [12]	rKR95	*L. donovani*	Kinesin-related protein	Bangladesh	Not applicable; see text.	GI112293604

^a^Strain details and GenBank accession reported in [16]

## Methods

GenBank searches of sequences homologous to the archetypal diagnostic antigen rK39 identified publications from which the matched sequences were first reported. Publications using subsequent novel rK nomenclature were identified from their listing on NCBI PubMed.

## Results

**Fig. 1:**
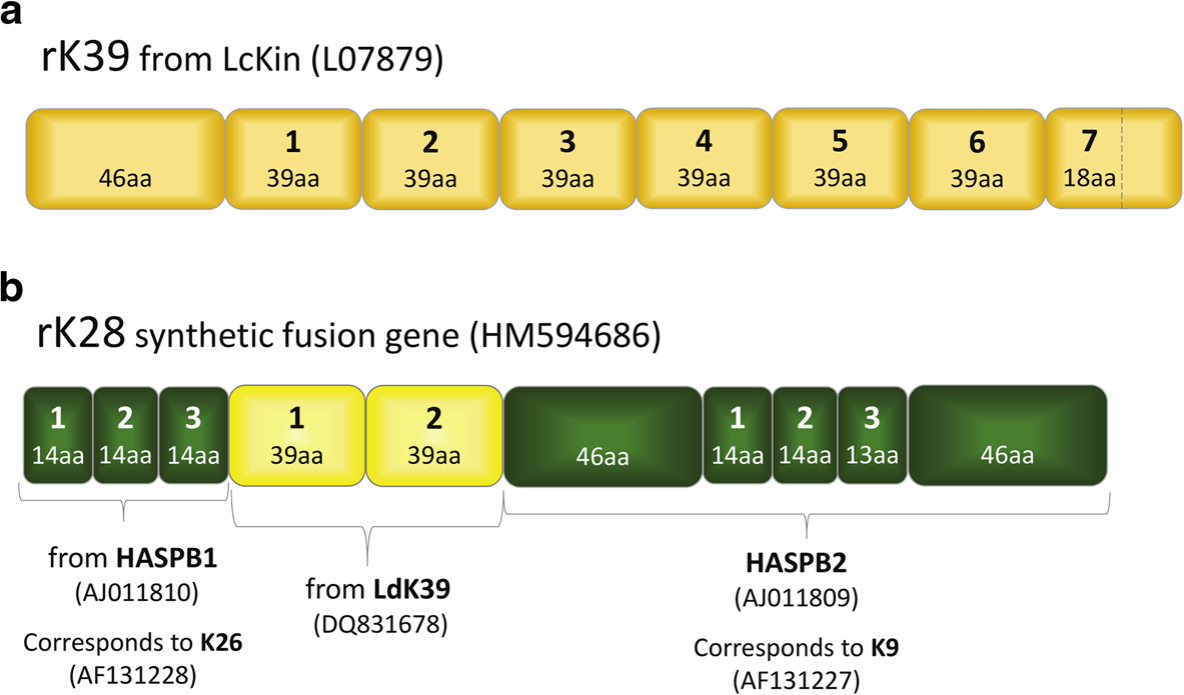
Schematic representations of VL diagnostic antigens with GenBank accession numbers: **a** rK39; **b** synthetic fusion rK28 and its components. Kinesin-derived sequences are depicted in *yellow* shades, to indicate their different species origin; HASPB sequences depicted in *green*. Numbering in bold refers to the order of the respective repeat region in the parent protein. *Abbreviation*: aa, amino acid

In 1993, in a seminal publication, Burns et al. [2] used a genomic library from a Brazilian strain of *L. infantum* (syn. *chagasi*) to identify a kinesin-related gene having high specificity and sensitivity in VL serology. A fragment of this gene, encoding a 46 amino acid region followed by 6.5 × 39 aa repeats, was expressed as a recombinant protein in *E. coli* and called rK39 (Fig. 1), where the prefix letter r stands for recombinant.

The following year, a previously identified lipophosphoglycan-associated protein in *L. donovani* entered the literature as KMP-11 (kinetoplastid membrane protein [3]). We have included this antigen in this review because it has been often reported in the literature, but in this case the K initial was not given to maintain a K nomenclature.

In 1999, Alce et al. reported the identification and antigenicity of two gene products, named HASPB1 and HASPB2 (for hydrophilic acylated surface protein B), from an Ethiopian strain of *L. donovani* [4]. However, in the same year and the same journal, Bhatia et al. independently reported the characterisation of two hydrophilic antigens, from *L. infantum* (syn. *chagasi*), which they named K9 and K26 [5]. There are two crucial considerations regarding the description of these latter two antigens: (i) the authors adopted the letter ‘K’ prefix in order to maintain consistency with K39; (ii) their report also refers to the identification in GenBank of homologous sequences from *L. donovani*, namely those identified by Alce and colleagues, and thus K9 corresponds to HASPB2, and K26 to HASPB1.

In 2006 and 2007, the first kinesin sequences from South Asian (Indian) and East African (Sudanese) *L. donovani* (Ld) strains were reported as Ld-rKE16 and LdK39, respectively [6, 7]. Note that the ‘KE’ in Ld-rKE16 refers to the given strain name, and not the two-letter abbreviation for Kenya. The first two 39 amino acid repeats of the Sudanese homologue LdK39 were later incorporated into a synthetic gene, where they were flanked by the repeat sequences of HASPB1 and the whole open reading frame of HASPB2, identified by Alce and colleagues (Fig. 1). This new construct was named rK28, further maintaining the letter K nomenclature of *L. donovani* antigens useful in diagnostic VL serology, and the prefix ‘r’ denoting a recombinant protein [8].

The K prefix has also been used for more reported antigens, namely rKRP42 [9], rKLO8 [10], rK18 [11] and rKR95 [12]. rKRP42: derived from strain DD8, which was described by the authors as being from Bangladesh but is listed as World Health Organisation (WHO) reference strain MHOM/IN/80/DD8 originating from India [13, 14]. rKLO8: described by the authors as deriving from Sudanese strain Lo8 (lower case letter o) without full WHO code, but a strain with WHO code MHOM/IN/??/Lo8 [sic], had previously been reported also with a lower case letter o but stating an Indian origin [15]. rK18: no sequences or derivations were given. rKR95: identified from mass spectrometry data of Bangladeshi serum and urine and given an accession number (see Table 1). However, searching this number in GenBank (without the sequence identifier prefix ‘GI’) retrieved the entry for LdK39, which was submitted by Gerald et al. 2007 and the sequences of which have been used for the kinesin repeats of rK28 [7, 8].

## Conclusions

Our intention here is to document the reporting of VL diagnostic antigens using the letter ‘K’ or ‘rK’ prefix nomenclature. We suggest that, to avoid confusion, the use of such nomenclature for future antigens should either provide the logical derivation of the term, for example, indicating the origin, any known function or protein family or that the designation is entirely empirical.

## Abbreviations

HASP: Hydrophilic acylated surface protein
VL: Visceral leishmaniasis
WHO: World Health Organisation
